# Pulmonary-Renal Syndrome During COVID-19 Pandemic Revealed a Rare Case of Anti-GBM Disease

**DOI:** 10.31138/mjr.34.2.256

**Published:** 2023-06-30

**Authors:** Aglaia Chalkia, Christos Koutsianas, Emelina Stampolliou, Panagiota Giannou, Harikleia Gakiopoulou, Dimitrios Vassilopoulos, Dimitrios Petras

**Affiliations:** 1 Nephrology Department, Hippokration General Hospital, Athens, Greece,; 2 2nd Department of Medicine and Laboratory, Clinical Immunology - Rheumatology Unit, National and Kapodistrian University of Athens School of Medicine, Hippokration General Hospital, Athens, Greece,; 3 1st Department of Pathology, National and Kapodistrian University of Athens, School of Medicine, Athens, Greece

**Keywords:** anti-GBM disease, rapidly progressive glomerulonephritis, pulmonary haemorrhage

## Abstract

Anti-GBM disease is a rare, life-threatening small vessel vasculitis caused by circulating anti-GBM antibodies resulting to rapidly progressive glomerulonephritis and/or pulmonary haemorrhage. The gold standard for the diagnosis is the renal biopsy with the pathognomonic finding of linear deposition of IgG along the glomerular capillaries. Early diagnosis and intervention are key determinants of the response to therapy and long-term prognosis of these patients. However, during COVID-19 pandemic recognizing a pulmonary-renal syndrome caused by autoimmune diseases has become challenging. Herein, we aimed to describe a rare case of anti-GBM disease with pulmonary haemorrhage and rapidly progressive glomerulonephritis in a young man in a tertiary referral hospital in Greece, while COVID-19 pandemic was at its peak. Although the patient presented high level of creatinine and crescents, the early diagnosis and start of treatment resulted to favourable renal prognosis.

## INTRODUCTION

Anti-GBM disease is a rare, life-threatening small vessel vasculitis. Circulating antibodies are directed against glomerular basement membrane (GBM), resulting to rapidly progressive glomerulonephritis as well as alveolar basement membrane (ABM) resulting to alveolar hemorrhage.^1^ Anti-GBM nephritis accounts for approximately 15 percent of all cases of crescentic glomerulonephritis according to case series.^2^ Younger patients usually present pulmonary haemorrhage, compared to older people who more often present only nephritis. However, <10% of the patients will present only pulmonary haemorrhage. The principal target for the anti-GBM antibodies is the NC1 domain of the alpha-3 chain of type IV collagen (alpha-3[IV] chain), one of six genetically distinct gene products found in basement membrane collagen and these antibodies are usually IgG.^3^ Recently, it has been described a rare variant of anti-GBM disease, described as “atypical anti-GBM nephritis” without pulmonary haemorrhage and undetectable antibodies.^4^ During the COVID-19 pandemic, recognising a pulmonary-renal syndrome caused by autoimmune diseases has become challenging. Herein, we aimed to describe a rare case of anti-GBM disease with pulmonary haemorrhage and rapidly progressive glomerulonephritis in a young man in a tertiary referral hospital in Greece, while COVID-19 pandemic was at its peak.

## CASE PRESENTATION

A 21-years-old healthy male and smoker from Iran was presented at the emergency room complaining of fever and fatigue. Physical examination showed peripheral capillary oxygen saturation (Sp02) was 98%, blood pressure 120/80 mmHg, heart rate of 100 beats per minute, and body temperature 38 °C. A chest x-ray showed diffuse alveolar pulmonary infiltrates and the chest computed tomography scan showed ground-glass opacities. As he was admitted during the period of COVID-19 pandemic, he was first transferred to the infectious diseases’ unit, and real-time reverse transcriptase-polymerase chain reaction (RT-PCR) for SARS-CoV-2 on nasal and oropharyngeal swabs test were performed and antibiotics were given.

The laboratory results at the time of admission are detailed in **Table 1**. The patient presented with Acute kidney injury (AKI) stage 2 (according to the KDIGO classification, without previous baseline serum Creatinine) and severe anaemia (hb=6.9 g/L). To further evaluate the cause of AKI, a renal ultrasound was performed which showed normal-sized kidneys and morning urine test which showed microscopic haematuria (dysmorphic RBCs 75%) and proteinuria nephrotic range (ACR 300mg/g). Although, the patient did not complain of breathless or haemoptysis, he started pulses of intravenous glucocorticoids considering as a possible pulmonary-renal syndrome. He remained in the infectious diseases’ unit for 2 days until two PCR for SARS-COV-2 proved negative and then transferred to the Nephrology Unit.

**Table 1.** Initial and post-treatment laboratory findings.

**Parameters**	**Initial value**	**Post 6month value**	**Normal ranges**
Hb	6.9	11.4	12–18 g/dL
urea	91	25	18–55 mg/dL
creatinine	2.1	1.1	0.72–1.25 mg/dL
Sodium	137	138	136–145 mmol/L
Potassium	5	3.5	3.5–5.1 mmol/L
Glucose	150	82	70–105 mg/dL
Calcium	8.7	9.2	8.4–10.2 mg/dL
Albumin	3.3	3.8	3.5–5 g/dL
AST	9	21	5–34 U/L
ALT	6	11	0–55 U/L
CRP, mg/L	3.25	1.13	0–5 mg/L
WBC count,	11.66	8.34	5.2–12.4 x x10e3/uL
Platelet count,	371	290	130–400 x x10e3/uL
Lymphocyte count,	0.97	2.22	0.9–5.2x x10e3/uL
IgG	549	654	700–1600 mg/dL
IgM	27	78	40–230 mg/dL
IgA	102	116	70–400 mg/dL
C3	110	152	75–180 mg/dL
C4	35	55	10–40 mg/dL
Proteinuria	3.320	930	<150 mg/24h
Urine RBC/HPF	100–120	50–70	0–1
Urine WBC/HPF	18–20	2–3	0–5

Hb: hemoglobulin; AST: Aspartate transaminase; ALT: alanine aminotransferase; CRP: C-reactive protein; WBC: White blood cells; IgG: Immunoglobulin G; IgM: Immunoglobulin M; IgA: Immunoglobulin A; C3: complement 3; C4: complement 4; RBC: red blood cells; WBC: white blood cells; HPF: high power field.

**Figure 1.**
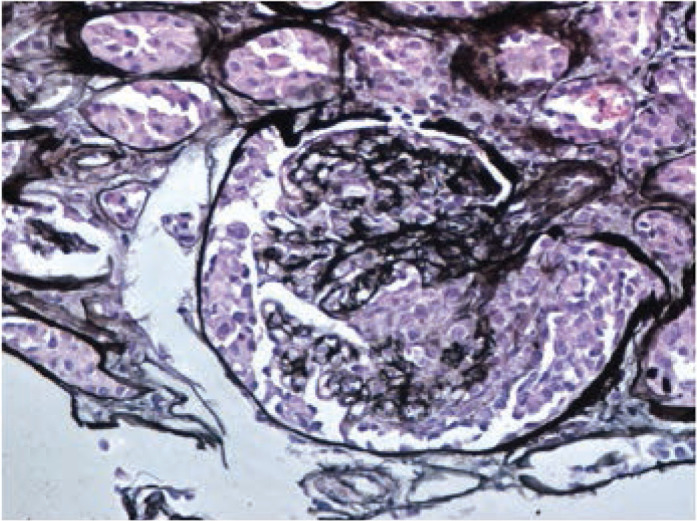
Light microscopy-cellular crescent formation (silver stain, original magnification x 200).

**Figure 2.**
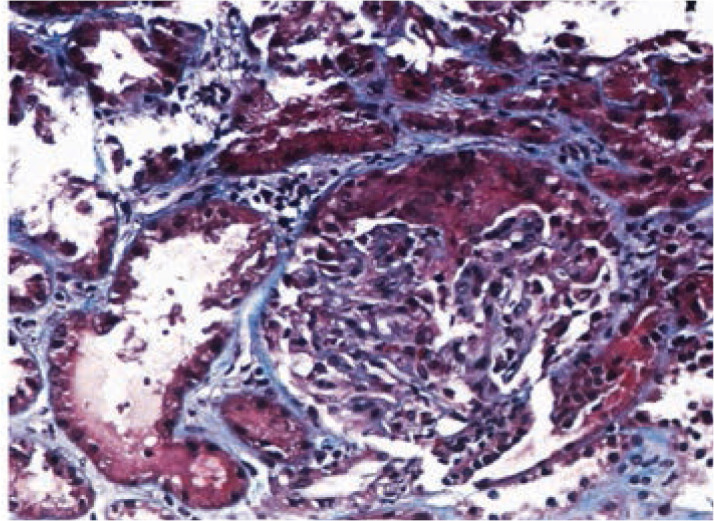
Light microscopy-cellular crescent formation (Masson stain, original magnification x200).

**Figure 3.**
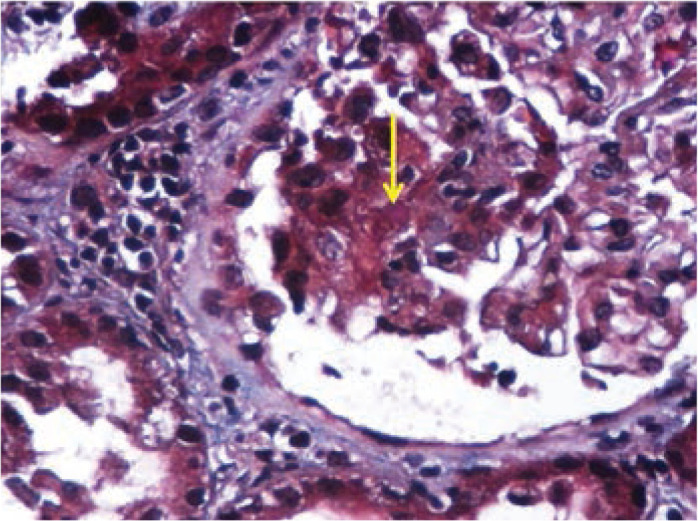
Light microscopy-cellular crescent formation and segmental necrosis (yellow arrow) (Masson stain, original magnification x 400).

**Figure 4.**
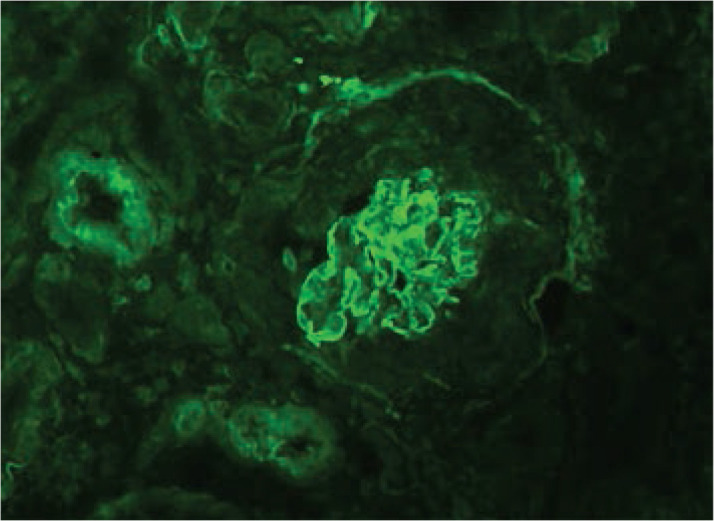
Immunofluorescence-linear deposition of IgG along the glomerular capillaries in a crescent formation.

After two days, the creatinine level was further increased at 4.5mg/dl. The fourth day, a kidney biopsy was performed to determine the cause of a possible crescentic glomerulonephritis. In the meantime, further investigation was performed to exclude possible autoimmune diseases including, serologic testing for hepatitis B, hepatitis C, and HIV, cryoglobulins, serum complement for C3 and C4, antinuclear antibody, anti-double-stranded DNA, ANCA, anti-GBM antibodies. Although the patient had already received three pulses of corticosteroids, he presented dyspnoea with low SpO2 and further reducing hemoglobulin, without clinical sign of haemoptysis and therefore plasmapheresis regimen was added, as rescue treatment for possible pulmonary haemorrhage. According to the biopsy results, light microscopy revealed crescentic glomerulonephritis with 92% cellular crescents, 8% normal glomeruli and the immunofluorescence demonstrated the virtually pathognomonic finding of linear deposition of IgG along the glomerular capillaries and occasionally the distal tubules, indicating anti-GBM nephritis. (**Figures 1–4**) The anti-GBM antibodies were found positive [level 86 (positive>30 ELISA method)] whereas the antineutrophil cytoplasmic antibodies were negative.

Overall, the patient received intravenous pulses with cyclophosphamide and plasmapheresis regimen (prescription: 4 litres exchanges with fresh frozen plasma daily for 15 procedures), and the anti-GBM antibodies became negative (10 days after the initiation of the plasmapheresis). He continued cyclophosphamide for 3 months and started tapering of glucocorticoids for 6 months. We continued to monitor anti-GBM antibody levels monthly for 6 months and they remained negative After, 6 months the patient presents normal renal function with creatinine level 0.8mg/dL, persistent microscopic haematuria, proteinuria <1gm/24H and negative anti-GBM antibodies.

## DISCUSSION

This case report highlights a rare case of renal-pulmonary syndrome, which every physician should consider in patient with evidence of pulmonary haemorrhage, even without clinical sign of haemoptysis, and rapidly progressive glomerulonephritis. Especially, during the period of COVID-19 when the most patients with fever or dyspnoea and ground glass in CT lung concurrently quite often with AKI would be considered as SARS-CoV-2 infection, further evaluation of the cause of AKI with urine assessment should be performed and in cases with active urine sediment high suspicion of anti-GBM, amongst other autoimmune diseases such as ANCA associated vasculitis, IgA or cryoglobulinemic vasculitis, SLE as well as infection-related glomerulonephritis, is essential. The gold standard for the diagnosis is the renal biopsy with the pathognomonic finding of linear deposition of IgG along the glomerular capillaries, because the anti-GBM antibodies could also be present in other conditions, for example ANCA associated vasculitis or could be false negative dependent on the commercial ELISA assays or as a rare variant described as “atypical anti-GBM nephritis” and therefore renal biopsy should not be delayed.

**Figure 5.**
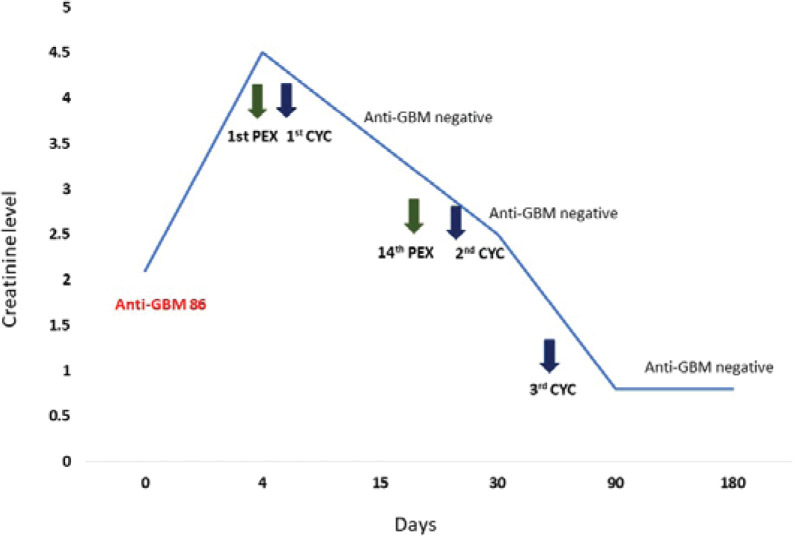
Level of creatinine and anti-GBM antibodies over time.

According to the literature, the majority patients with anti-GBM are smokers, and the most common symptom that leaded them to the medical consultation was fatigue at 33%, while fever, dyspnoea, or haemoptysis were found under 10%.^5^ Likewise, the systemic complaints and signs when are present, are usually experienced for only a few weeks. In our case the systemic symptoms (fever and fatigue) had driven the patient to the emergency without complaining of haemoptysis or breathless. Furthermore, while our patient did not present dual ANCA positivity, it could be found in one third of the patients with anti-GBM disease.

Case series with anti-GBM disease revealed that creatinine level >5.6mg/dL, percentage of crescents >75%, or lower percentage of normal glomeruli have been proved as negative prognostic factors for renal prognosis. ^6,7^In contrary, even though our patient presented high level of creatinine at diagnosis (peak level of 6 mg/dL) and significantly increased percentage of crescents in the renal biopsy (>90%) and few normally glomeruli, he presented favourable renal prognosis (at 6 months normal renal function). Presumably, this may reflect the immediate start of the combination treatment (immunosuppressive and plasmapheresis) to our patient and points out the unmet need for high suspicion given that anti-GBM is a life-threatening disease, and the usage of plasmapheresis has been found to be beneficial for the patients [HR 0.29 95% CI (0.08–0.98), p=0.046].^5^

Large prospective studies have not been carried out due to the rarity of the cases. Immunosuppressive treatment consisted of corticosteroids and cyclophosphamide have been used for 3 months, as anti-GBM disease has described as monophasic disease with rare relapses.^8^ Rituximab has been used in resistant disease.^9^ Our patient received 15 plasma exchanges and 3 doses of intravenous cyclophosphamide, and the anti-GBM antibody levels remained consistently negative after the tenth day of plasmapheresis.

Consequently, most patients with anti-GBM disease present clinical features of rapidly progressive glomerulonephritis and around the half of them with concomitant alveolar haemorrhage. Anti-GBM antibodies should be work out in every clinical suspicion, and kidney biopsy should not be delayed. Therefore, the diagnosis of anti-GBM disease could be challenging and high suspicion of the physicians is an unmet need, because early diagnosis and intervention could drive to a favourable outcome.
